# Geographic-Scale Coffee Cherry Counting with Smartphones and Deep Learning

**DOI:** 10.34133/plantphenomics.0165

**Published:** 2024-04-03

**Authors:** Juan Camilo Rivera Palacio, Christian Bunn, Eric Rahn, Daisy Little-Savage, Paul Günter Schmidt, Masahiro Ryo

**Affiliations:** ^1^ Leibniz Centre for Agricultural Landscape Research (ZALF), Müncheberg, 15374, Germany.; ^2^ Alliance of Bioversity International and CIAT, Rome, 00153, Italy.; ^3^ Brandenburg University of Technology Cottbus-Senftenberg, Cottbus, 03046, Germany.; ^4^ Producers Direct, London, E2 8EX, UK.

## Abstract

Deep learning and computer vision, using remote sensing and drones, are 2 promising nondestructive methods for plant monitoring and phenotyping. However, their applications are infeasible for many crop systems under tree canopies, such as coffee crops, making it challenging to perform plant monitoring and phenotyping at a large spatial scale at a low cost. This study aims to develop a geographic-scale monitoring method for coffee cherry counting, supported by an artificial intelligence (AI)-powered citizen science approach. The approach uses basic smartphones to take a few pictures of coffee trees; 2,968 trees were investigated with 8,904 pictures in Junín and Piura (Peru), Cauca, and Quindío (Colombia) in 2022, with the help of nearly 1,000 smallholder coffee farmers. Then, we trained and validated YOLO (You Only Look Once) v8 for detecting cherries in the dataset in Peru. An average number of cherries per picture was multiplied by the number of branches to estimate the total number of cherries per tree. The model's performance in Peru showed an *R*^2^ of 0.59. When the model was tested in Colombia, where different varieties are grown in different biogeoclimatic conditions, the model showed an *R*^2^ of 0.71. The overall performance in both countries reached an *R*^2^ of 0.72. The results suggest that the method can be applied to much broader scales and is transferable to other varieties, countries, and regions. To our knowledge, this is the first AI-powered method for counting coffee cherries and has the potential for a geographic-scale, multiyear, photo-based phenotypic monitoring for coffee crops in low-income countries worldwide.

## Introduction

Coffee is one of the most widely consumed beverages, produced across more than 70 tropical countries, involving 12 million smallholder farmers globally [1]. Coffee production faces the risk of declining yields and quality due to drastic changes in temperature and precipitation [2–4], frequent pest and disease outbreaks [5], unstable selling prices, and high input costs, such as fertilizers and herbicides. Furthermore, it is projected that up to 50% of the land suitable for coffee cultivation could be lost globally by 2050 due to climate change [6]. Therefore, it is crucial to monitor coffee crops at large spatial scales to cope with climate change with phenotyping.

Recent advances in agricultural digitalization, including remote sensing, mechanistic simulation, and artificial intelligence (AI), have the potential to enable large-scale, accurate phenotypic monitoring for various crop types while addressing the high cost of measuring equipment, the cost of labor, the high complexity of the models or methods, the unavailability of Earth observations, and the lack of historical plant information including weather, soil, and management practices. For instance, yield prediction can utilize cameras equipped with RGB, red-edge, and multispectral bands, as well as unmanned aerial vehicles (UAVs). However, such devices are often too expensive for smallholder farmers, particularly in low-income countries. Recent studies have explored mapping in coffee using remotely sensed imagery. However, it remains challenging to apply remote sensing techniques because coffee trees are difficult to identify visually from space due to intercropping, frequent dense cloud coverage, and tree canopy cover [7].

To date, statistical modeling approaches have been the most popular method for estimating coffee crop productivity [8]. For example, [9] developed a method to count the number of cherries per tree by dividing the plant into 4 quadrants and then counting the nodes and cherries per node for each quadrant. Despite its high accuracy, it is time-consuming to design the division of the plant into quadrants for each individual plant. Moreover, it requires the condition of nonsteep and nonmuddy slopes in the field, which is impractical in many locations [8]. Other studies [10–12] propose productivity prediction using a sample of the productive lateral branches and an estimate of the quantity per lateral. Furthermore, [13] introduced a method based on genomic information for coffee production and [14] with agrometeorological information. Despite the high accuracy of these models, limitations persist concerning data acquisition and the breadth of available data.

In addition to statistical modeling, there is a growing number of studies employing machine learning methods. For instance, [15] developed a model based on an extreme learning machine and random forest algorithms to link soil fertility properties with Robusta coffee production in Vietnam, achieving a coefficient of determination (*R*^2^) of 0.60. In another study, [16] utilized UAV-taken aerial images of 144 trees in Mina Gerais. They employed 5 algorithms: linear support machines, gradient boosting regression, random forests, partial least square regression, and neuroevolution of augmenting topologies, the latter proving to be the most effective, exhibiting a mean absolute percentage error of 32%. Similarly, [17] proposed segmentation and a convolutional neural network (CNN) for the *Castillo* coffee variety using mobile images, achieving an *R*^2^ of 0.59. While these studies demonstrate the promising potential of AI applications for coffee production, they typically focus on one variety in a single country and require well-trained personnel for image acquisition. Collectively, none of the existing methods are suitable for estimating coffee crop yields on hundreds or thousands of smallholder farms at large spatial scales (i.e. scalability). A citizen science approach to data collection could address this scalability issue [18].

We aim to develop a geographic-scale, low-cost method to monitor the number of coffee cherries with the help of local farmers by using pictures captured on their mobile phones. To achieve this, we developed a relatively simple field sampling protocol that local farmers can follow individually without formal training. We collected images and applied the You Only Look Once (YOLO) v8 object detection method. Our approach garnered support from thousands of local coffee farmers, facilitating data collection across multiple regions in Peru and Colombia. Importantly, we evaluated the model's generalizability and transferability across countries and diverse coffee varieties, a novel effort not previously undertaken.

## Materials and Methods

### Study site

**Fig. 1.**
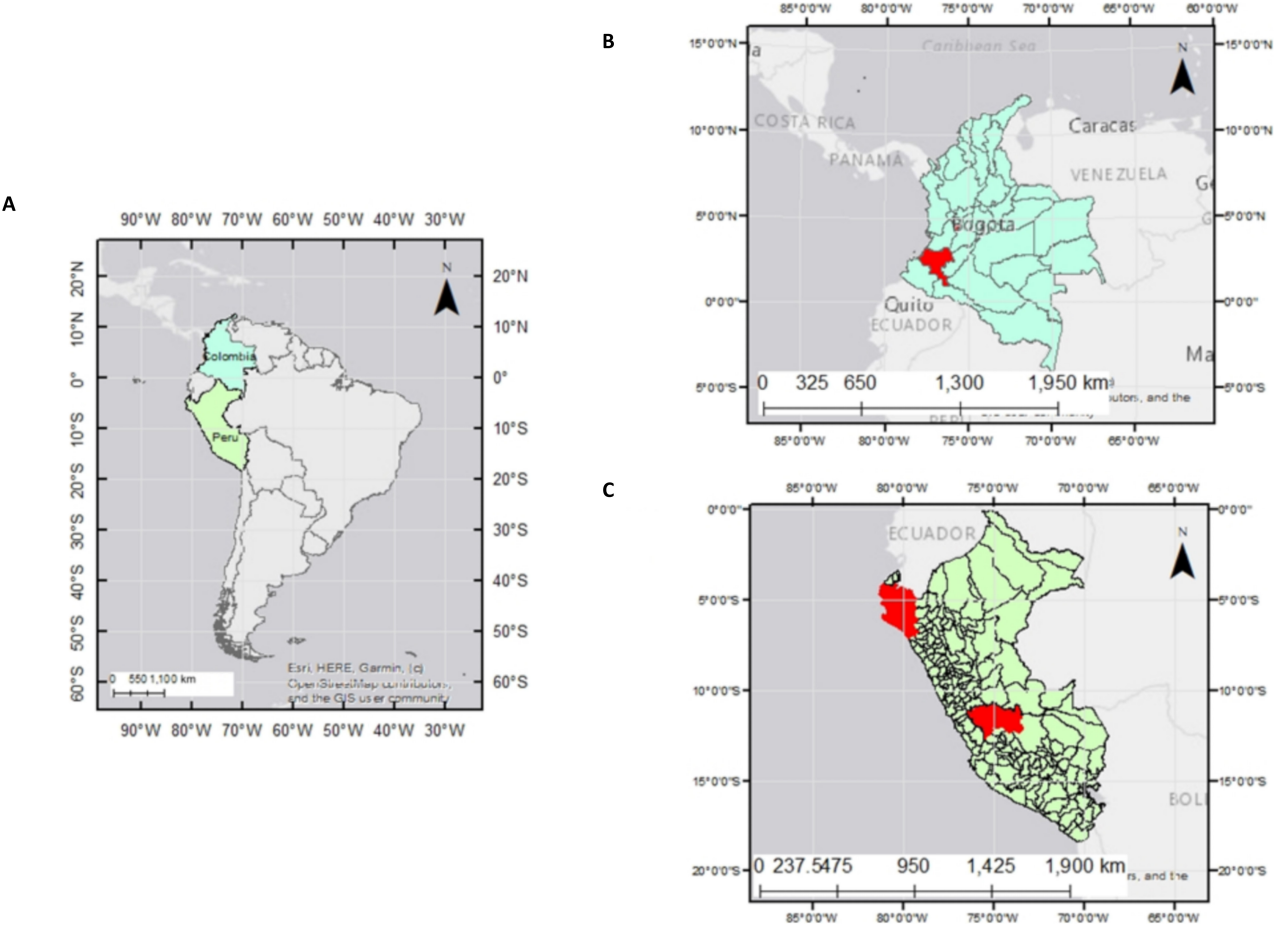
Location of the target regions. (A) Target countries: Colombia (light blue) and Peru (light green). (B) Génova, Cajibío, El tambo, Morales, Piendamó, and Popayán in Colombia (red). (C) Chinchaque, Chirinos, Cañariz, Lalaquiz, Pongoa, and San José Lourdes in Peru (red). We developed the model in Peru and tested it in both Peru and Colombia.

The target regions of this study (Fig. 1) are the northern and central parts of the coffee districts in Peru (Chinchaque, Chirinos, Cañariz, Lalaquiz, Pongoa, and San José Lourdes; latitude–longitude of 5°–6°S, 6°–7°W) and the south-western and western parts of the coffee municipalities in Colombia (Génova, Cajibío, El tambo, Morales, Piendamó, and Popayán; 2°–4°N, 75°–77°W).

The target districts in Peru cover elevational gradients and produce highly heterogeneous environments with variations in soil types. The maximum monthly temperature ranges between 20 °C (July) and 34 °C (March), while the minimum monthly temperature varies between 14 °C (July to August) and 23 °C (February) [19] (region A in Fig. 1). In contrast, the target region in Colombia is humid and semihumid. Rainfall exhibits a bimodal pattern, peaking in April (120 mm) and October (110 mm). The average temperature hovers around 18.1 °C (October to November) and 21 °C (June to August) [20] (region B in Fig. 1). These 2 target regions exhibit distinct weather patterns; Colombia tends to be cooler and rainier than Peru.

### Sampling campaign with local farmers

Data collection involved a survey of 977 farmers in Colombia (300 farmers) and Peru (677 farmers). The majority of farmers surveyed (69% in Peru and 77% in Colombia) had a coffee area of less than 2 hectares (ha). A smaller percentage, 19% in Colombia and 28% in Peru had a coffee area between 2 and 5 ha, and only a fraction (3% in Colombia and 2% in Peru) had a coffee area greater than 5 ha. In addition, the majority of surveyed locations in Colombia (88%) were located at elevations between 1,500 and 2,000 m above mean sea level (A.M.S..L), whereas in Peru, most (61%) were located at elevations between 1,000 and 1,500 m A.M.S.L.

This study was focused on Arabica coffee (*Coffea arabica* L.). In Peru, the most common variety was *Catimor cogollo verde* 70%, followed by *Catimor cogollo morado* (8%), *Caturra* (1%), and *Tipica* (4%). Other varieties accounted for the remaining 17%. In Colombia, the majority of surveyed farmers used *Castillo* (90%), with small percentages of *Supremo* (4%), *Variedad Colombia* (1%), and other varieties (5%). In terms of tree age, 56% of the trees in Peru were between 3 and 7 years old, 39% were between 7 and 14 years old, and 5% were between 14 and 21 years old. In Colombia, 88% of the trees were between 3 and 7 years old, 12% were between 7 and 14 years old, and only 0.03% were between 14 and 21 years old.

### Sampling protocols with a pictured-based model and manual measurement

We received help from 53 survey personnel (hereafter, enumerators), who visited the local farmlands and took pictures of coffee trees through local partners during the cherry growing season, from March to August 2022 in Peru and from March to November 2022 in Colombia. The mobile pictures were collected using the enumerators' mobile devices. In total, 2,968 trees were investigated, yielding a collection of 8,904 pictures. Specifically, 2,450 trees were in Colombia and 518 in Peru, contributing to 7,350 and 1,554 pictures, respectively. The sampling protocol was designed as follows.

Nine trees were selected using a random sampling method per each coffee crop field. For each tree, 1 branch was randomly selected from each of the upper, middle, and lower positions (i.e., 3 branches per tree), and 1 photo was taken per branch. When taking a photo, the enumerators were instructed to adhere to the following guidelines: (a) capture photos during daylight hours, specifically between 6 AM and 6 PM; (b) focus on as many cherries as possible; (c) avoid moving the camera post-capture to prevent picture distortion; and (d) choose an angle that prevents direct sunlight from shining on the camera lens.

We attempted to capture the most cherries on each branch using a mobile phone image because our model uses this information to estimate the total number of cherries. Earlier studies have explored cherry detection using mobile images, employing computer vision techniques as segmentation [17], which is effective primarily for red cherries against green leaves, and incorporating machine learning and deep learning algorithms [21]. In this study, we proposed a protocol for photographing only 3 branches, rather than the entire tree, in order to capture as many cherries as possible.

The protocol was used to evaluate the reliability of using mobile images to replace the manual counting of cherries on branches. To assess the accuracy of our approach, we manually counted the cherries on the respective branches. The total number of cherries per tree was measured as follows: For each selected branch, the enumerator manually counted all cherries. An average number of cherries was calculated from the manually counted cherries of the upper, middle, and lower branches. This average was multiplied by the total number of productive branches on the tree, also counted manually. The product of this calculation was the total number of cherries per tree.

The images were taken using a variety of commercially available mobile phones owned by the enumerators. The most popular brands were Xiaomi, Samsung, and Motorola, with the Xiaomi M2006C3LG model being the most popular mobile. These mobiles capture images in RGB color format as JPEG format and are equipped with internal Global Positioning System capabilities. The majority of the 768 × 768 and 1,024 × 1,024 images were taken without flash at resolutions between 1 and 12 megapixels.

### Images annotation

The mobile phone images were annotated using the PASCAL VOC format and the labeling graphical image annotation tool [22]. The length and width of the training images were rescaled to 640 pixels. The process of manual annotation was initiated after the acquisition of the images were captured by the enumerators in the coffee fields. We defined 3 categories for annotation: black cherries, red cherries, and green cherries. This categorization enables the model to recognize the primary colors of cherries throughout their growth cycle and detect them at any stage prior to harvest. A minimum bounding box is drawn around the cherry for each of these categories. In the case of occlusion, we followed the methodology proposed by [23], which recommends not labeling cherries if the occlusion is greater than 85% and the visible target area is less than 15%. When a cherry is obscured by others, the bounding rectangle should encompass the entire cherry, including any parts that are behind the others. The final annotated dataset consisted of 436 images with a total of 35,694 labeled cherries: 35,247 (98.7%) were labeled as green cherries, 342 (0.9%) as red cherries, and 105 (0.2%) as black cherries. As the cherries were mainly counted during the growth stage when the cherries are green, the occurrences of the other colors are uncommon in our data set.

### YOLO v8 model for cherry detection

CNNs were used for cherry detection. We used the state-of-the-art object detection network, YOLO v8 network [24]. This network is an advancement of the original YOLO framework [25] and previous versions [24,26–30]. YOLO is a real-time object detection framework that is designed for fast object detection and classification. It simplifies the detection task into a regression problem, converting image pixels directly into bounding box coordinates and class probabilities [25]. The framework uniquely performs object detection through a single CNN and uses the entire image to predict each bounding box.

During the training phase, YOLO divides an image into a 7 × 7 grid. If the center of an object falls within the boundaries of a grid cell, the cell is responsible for detecting the object. Each cell is responsible for predicting bounding boxes, confidence scores, and class probabilities. The confidence score is defined as follows (Eq. 1):

$$\text{Confidence score}=\mathrm{Pr}\left(\;\text{Object}\right)\times {\mathrm{IoU}}_{\text{true}}^{\text{pred}}$$ (1)

The confidence score represents the model's certainty that the bounding box contains an object and the accuracy of its prediction of the size and location of the box. If there is no object, then the confidence score is 0. Otherwise, the confidence score is equal to the intersection of the prediction and the ground truth. YOLO generates multiple bounding box predictions per cell and employs Non-Maximum Suppression to identify the most accurate bounding box. The Intersection over Union (IoU) metric evaluates the precision of object detection by calculating the overlap ratio between the predicted bounding box and the true bounding box. The IoU is defined as follows (Eq. 2):

$$\mathrm{IoU}=\frac{S_{\text{overlap}}}{S_{\text{union}}}$$ (2)

where *S_overlap_* is the area of intersection between the predicted bounding box and the true bounding box, and *S_union_* is the total area covered by both bounding boxes.

**Fig. 2.**
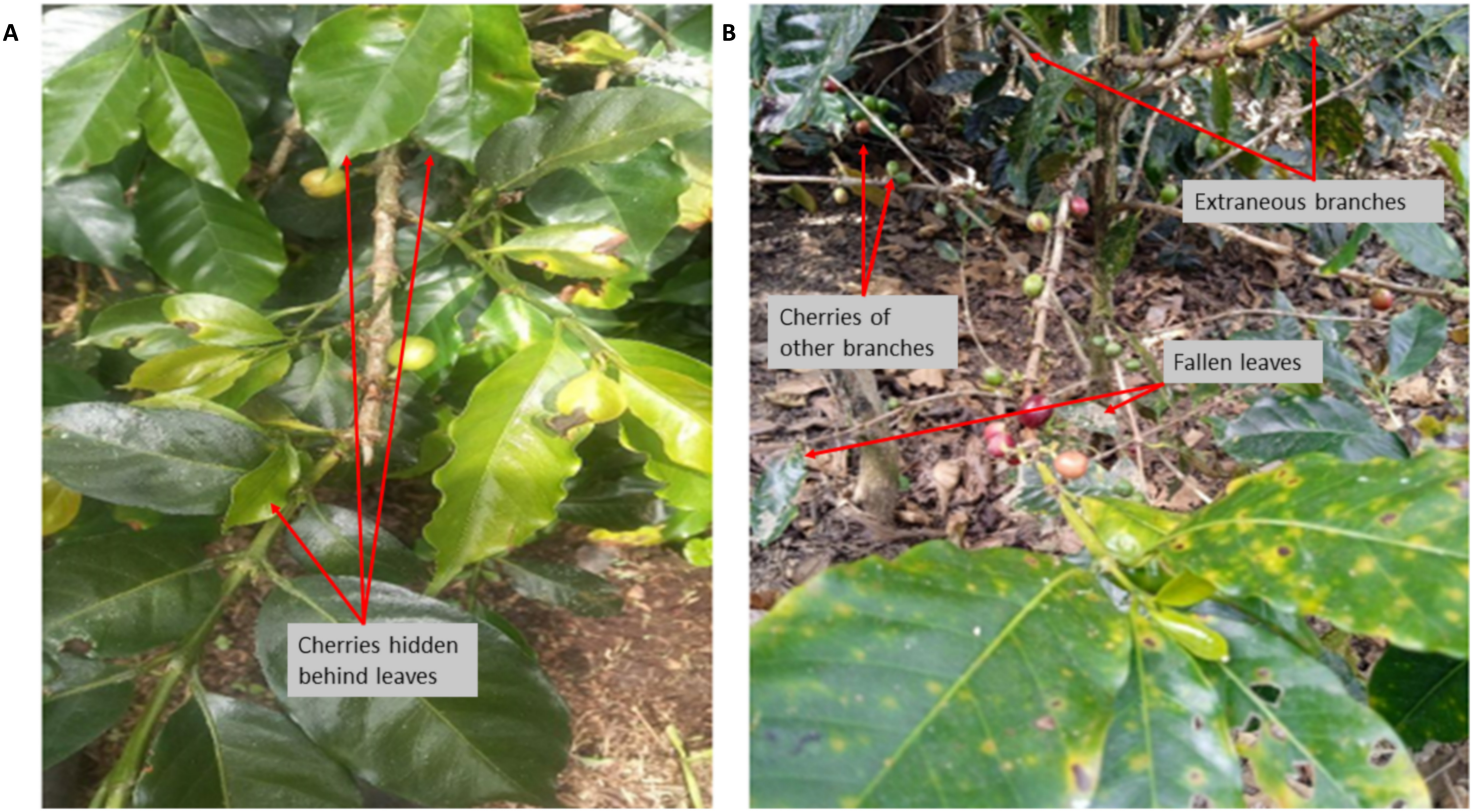
The interference of external objects in mobile images. (A) Large leaves blocking the view of the cherries on the branch. (B) Various external objects, including cherries from other branches, fallen leaves, and extraneous branches.

Despite their high accuracy, earlier versions of YOLO (v1 to v4) encountered difficulties with images acquired through remote sensing technologies such as drones. These challenges arise from variations in size, large coverage areas, and high object densities [24]. The high-density challenge particularly affects cherry detection in images acquired by mobile devices, where surrounding elements such as leaves and branches can interfere with accurate detection (Fig. 2).

In previous YOLO versions, YOLO v5 incorporates the Transformer Prediction Head [31], enhancing object localization accuracy in densely populated scenes. Additionally, it integrates the Convolutional Block Attention Module [32], improving the precision of identifying regions of interest across extensive areas [24]. This approach employs a self-training classifier to improve classification performance. YOLO v6 [27] features an extended backbone and neck design, whereas YOLO v7 [30] adopts a transformer architecture. Both versions aim to increase the accuracy and velocity compared to their predecessors.

YOLO v8 [33] utilizes an architecture similar to YOLO v5, with key modifications in the backbone, particularly in the module responsible for feature extraction. By incorporating an anchor-free model and a semantic segmentation model, YOLO v8 demonstrates versatility, effectively both object detection and semantic segmentation tasks [33].

In this study, the YOLO v8 detection model was adapted using the Ultralytics framework [29], which is built on top of PyTorch 1.7 [34]. The models were developed on a Windows 10 platform using Python 3.7.0 subroutines running on an Nvidia GeForce RTX 3080 GPU 1440 MHz.

In Peru, we categorized the collected data into 3 sets: training (80%, *n* = 346), validation (10%, *n* = 43), and testing (10%, *n* = 43). Each set served a specific purpose: model development, evaluation/selection, and performance testing, respectively. Additionally, Table 1 provides the network parameters.

**Table 1.** Initialization parameters of YOLO v8 dense network

Size of input images(pixels)	Batch	Momentum	Initial learning rate	Decay	Epochs
640 × 640	16	0.937	0.1	0.0005	100

In order to improve the detection accuracy of the model, the initial values of parameters were of a pretrained model developed by Ultralytics, which was trained on the COCO (Common Objects in Context) dataset [35]. The COCO dataset contains more than 330.000 images, featuring 2.5 million objects instances labeled across 80 different categories. To address the server's memory constraints, we adjusted input images to 640 × 640 and set the batch size to 16. We used 100 epochs to better analyze the training process. The momentum, initial learning rate, weight decay regularization, and the other parameters were set as default settings in YOLO v8. The model was trained after defining the training process.

Data augmentation artificially increases the size and diversity of the training set, allowing the model to reduce overfitting on image data [36]. The acquired images were preprocessed using data augmentation techniques, including brightness adjustment and geometric augmentation, specifically scaling, shearing, left-right flipping, and mosaic techniques. These techniques were applied to the cherry mobile images because they reflect real scenarios in coffee plantations. Brightness adjustment can eliminate the noise from ambient light or the low-resolution camera, and geometric augmentation allows for a more accurate representation of the shape and size of cherries, which can vary depending on growth stage, variety, climate, and agricultural practices [37]. Data augmentation was used in the training phase.

### Estimating the total number of cherries at the tree level from information about the number of cherries per branch

Our YOLO model is able to count the number of cherries on the selected branches. To estimate the number of cherries per tree, we also used data on the number of branches per tree, collected during the field campaign (see Sampling protocols with a pictured-based model and manual measurement). We, therefore, estimate the total cherries load per tree (*T*) in a coffee plant at any given moment as the product of the total number of productive branches (*P*) and the average of the number of cherries per branch (*C*), where *i* is the *i*th branch (*i* = 1 upper, *i* = 2 middle, and *i* = 3 lower positions) (Eq. 3).

$$T=P^{*}\;1/3^{*}\;\sum_{i=1}^{3}C_{i}$$ (3)

The following evaluation metrics were used for tree-level number of cherries estimation: the root mean square error, mean absolute error, mean absolute percentage error (MAPE), and *R*^2^. To confirm associations between the number of cherries in each branch, the total number of productive branches, and the total of cherries per tree during the validation phase, we conducted a correlation analysis.

Additionally, we utilized the bounding box predictions (box_loss) which measures the discrepancy between the prediction box and the ground truth bounding box [25]; the classification loss (cls_loss), representing the difference between the predicted and actual labels [38]; and the dynamic feature learning loss (dfl_loss), a loss function designed for the extraction of dynamic and discriminative features from data [39]. The model's performance was further evaluated using the mean average precision (mAP), which assesses the model's ability to detect and accurately localize boxes around objects within images [40], with a particular emphasis on up to 50 object detections per image (mAP50) or between 50 and 95 objects detections per image (mAP50-95), alongside recall and precision metrics.

## Results

### Description of the coffee tree

Table 2 presents the summary statistics of the number of cherries per tree based on manual counting. This includes the mean, minimum, maximum, standard deviation, skewness, and coefficient of variation (CV) for each country and variety. The average cherry counts were substantially higher in Peru, ranging from 554 to 1,824, compared to Colombia, where counts ranged from 174 to 455. Across all varieties, the maximum count was often more than 10 times the minimum. The CVs exceeding 40% indicate high variation in cherry counts among all varieties and countries. In summary, the sampling campaign captured a wide range, from 174 to 1,824 coffee cherries per tree, illustrating the diversity of cherry counts observed.

**Table 2.** Summary statistics of manually counted coffee cherries per tree. CV is the coefficient of variation.

Country	Coffee varieties	Mean (cherries per tree)	Min (cherries per tree)	Max (cherries per tree)	Standard deviation (cherries per tree)	Skewness	CV (%)	Sample size (total pictures)
Peru	*Catimor cogollo verde*	1,431.58	70.00	8,085.00	1,049.73	2.16	73.33	1,085
	*Catimor cogollo morado*	1,016.64	40.00	2,749.50	743.50	0.78	73.13	120
	*Caturra*	1,824.36	144.00	3,440.00	739.38	-0.20	40.53	28
	*Otros*	972.36	160.00	2,925.00	631.38	1.01	64.93	265
	*Typico*	553.86	153.00	1,012.50	233.68	0.09	42.19	56
Colombia	*Castillo*	413.18	3.33	4,982.67	435.08	2.62	105.30	6,623
	*Otros*	454.79	18.00	3,241.33	471.08	3.05	103.58	377
	*Supremo*	268.31	4.67	1,241.33	294.92	1.46	109.92	296
	*Variedad colombia*	174.04	5.33	912.00	271.65	1.98	156.09	54

**Fig. 3.**
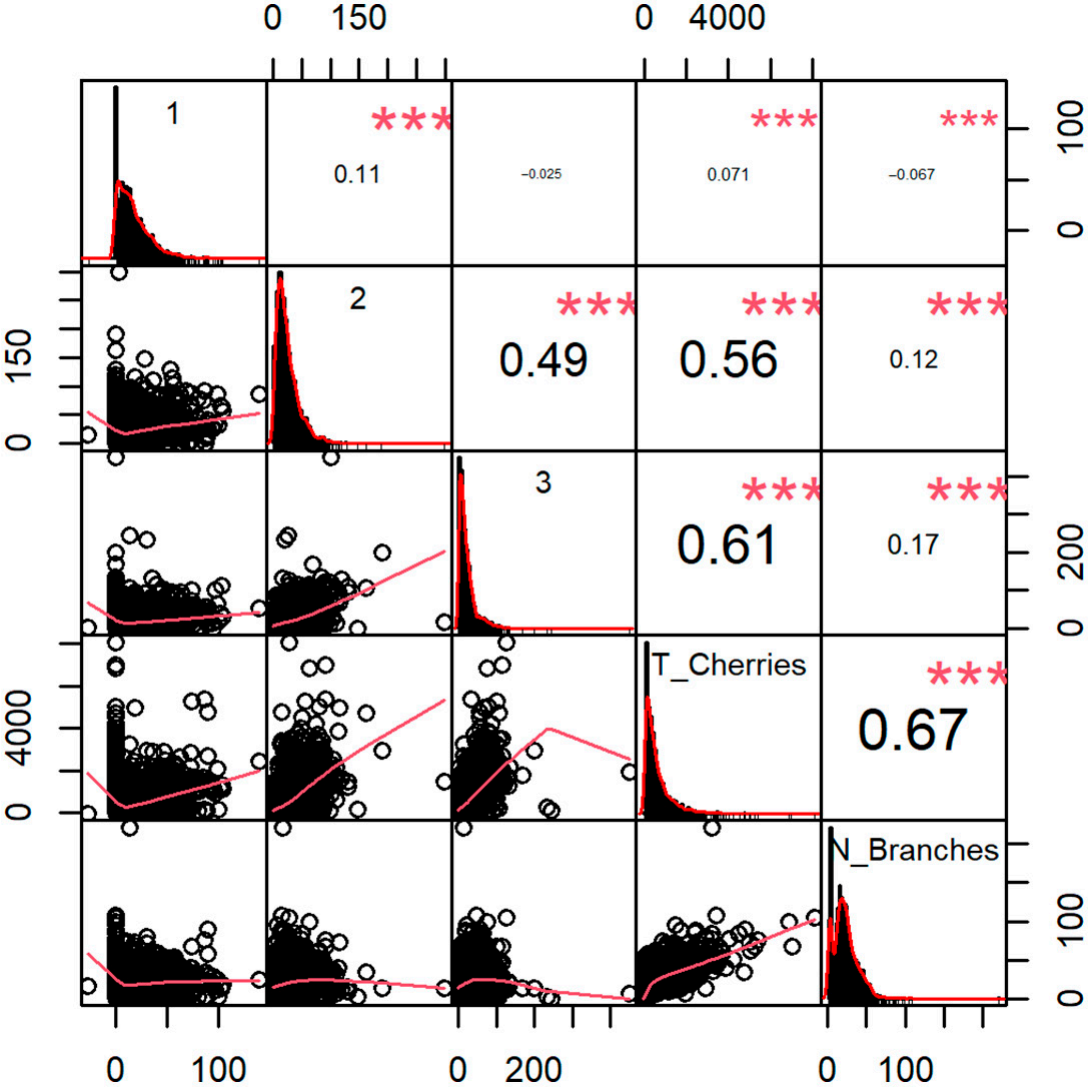
Correlation analysis of the number of coffee cherries. This figure shows the correlation between the number of total productive branches (N_Branches), the number of cherries on (1) the upper branch, (2) the middle branch, (3) the lower branch, and the total number of coffee cherries per tree (T_Cherries). Data from both Peru and Colombia are included in this analysis.

The number of productive branches demonstrates the strongest correlation with the total number of cherries at the tree level (*r* = 0.67, *P* < 0.001) (Fig. 3). Additionally, the numbers of cherries across different branch positions (top, middle, and bottom positions) were found to be correlated, though weakly (*r* = 0.11 for upper and middle, *r* = −0.025 for lower and upper, and *r* = 0.49 for middle and lower; *P* < 0.001 for all) (Fig. 3). This suggests that sampling from various heights is crucial for a more accurate estimation of the total number of cherries at tree level.

### YOLO v8’s performance for cherry detection

**Fig. 4.**
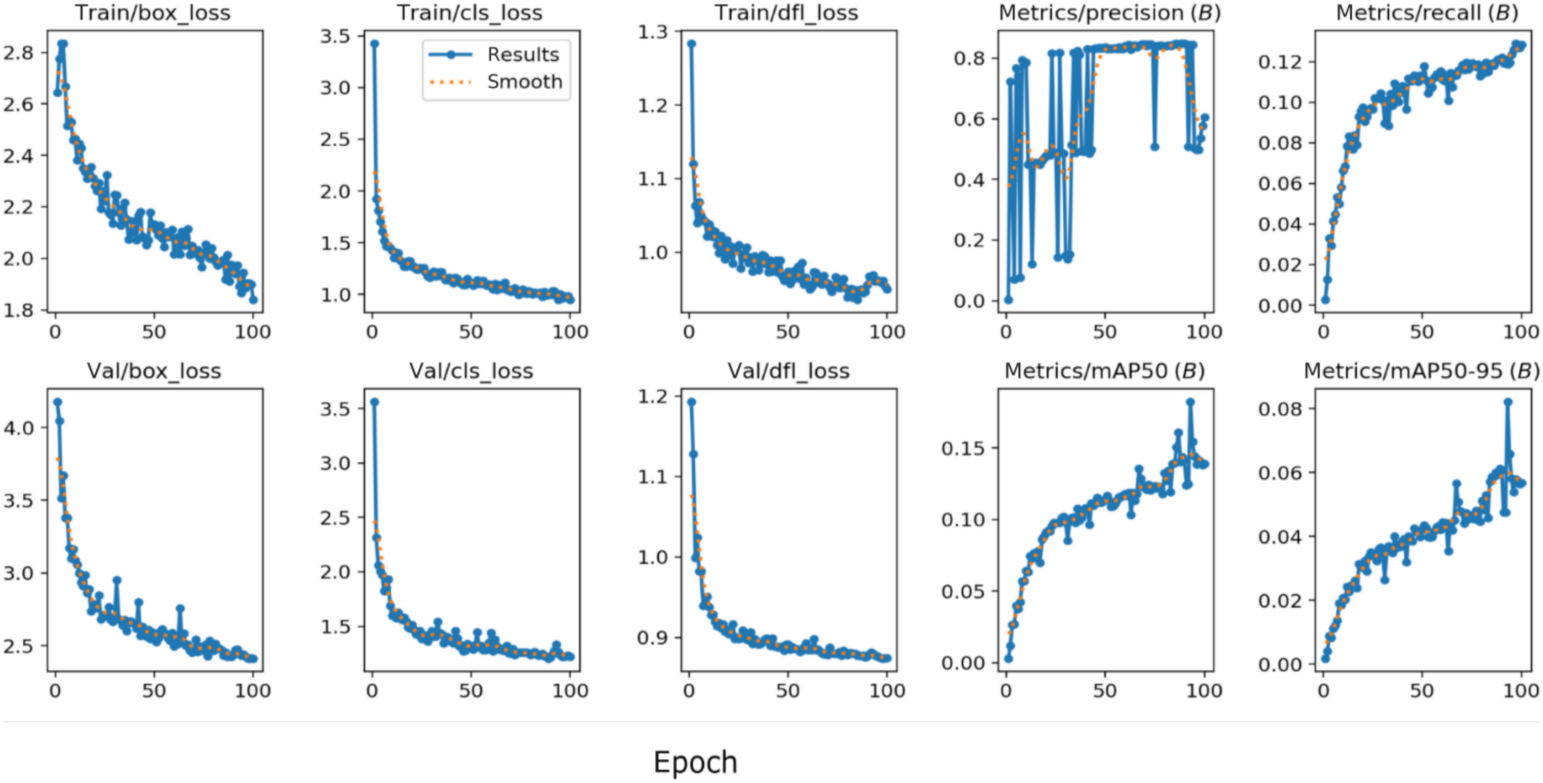
Loss and metric functions during training and validation phases with YOLO v8. It includes box_loss, cls_loss, dfl_loss, metric precision (B), and metric recall (B), applying IoU threshold *B* = 0.5.

YOLO v8 was trained with the images of coffee cherries (green, red, and black). The losses associated with the bounding box, classification, dynamic feature learning, precision, and recall metrics functions are shown in (Fig. 4).

The performance of object detection was characterized by a high precision of 0.85 and a low recall of 0.13 with threshold *B* set at an IoU of 0.5 (Table 3).

**Table 3.** Summary of metrics: Precision, recall, mAP50, and mAP50-95 for YOLO v8 at Epoch 100 with threshold *B* set at an IoU of 0.5

Epochs	Precision (*B*)	Recall (*B*)	mAP50 (*B*)	mAP50-95 (*B*)
100	0.85	0.13	0.18	0

### Model performance at branch level

**Fig. 5.**
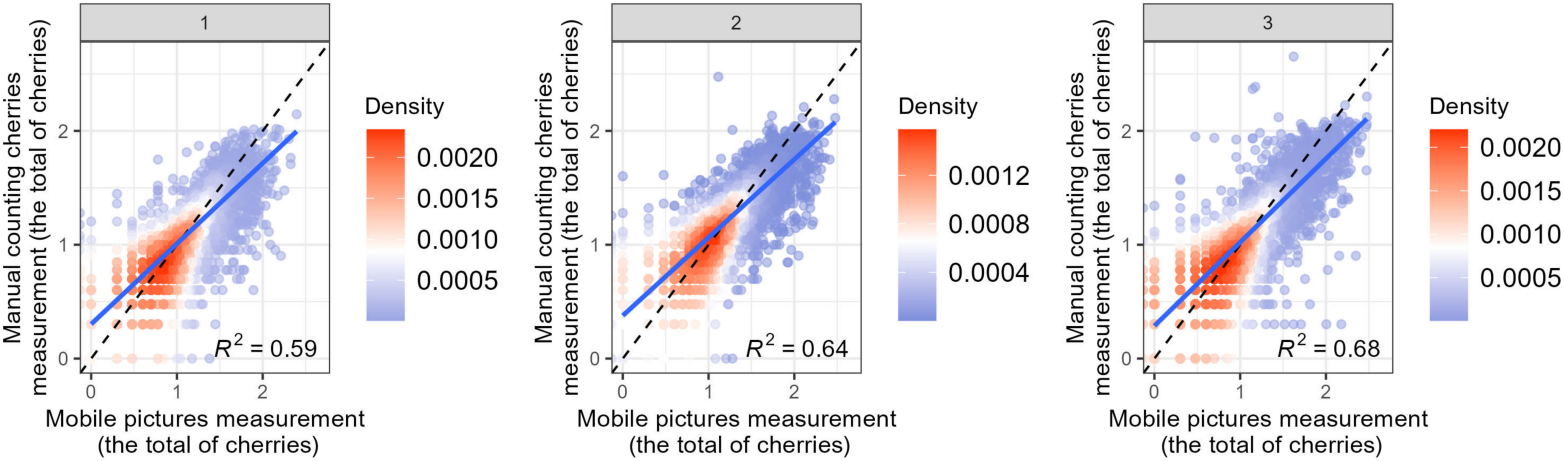
Comparison of manual counts and picture-based estimations. This figure displays the comparison between manually counted cherries and picture-based estimations for (1) upper branch, (2) middle branch, and (3) lower branch (*n* = 8,904 each). The axes are log10(X+1)-transformed.

**Fig. 6.**
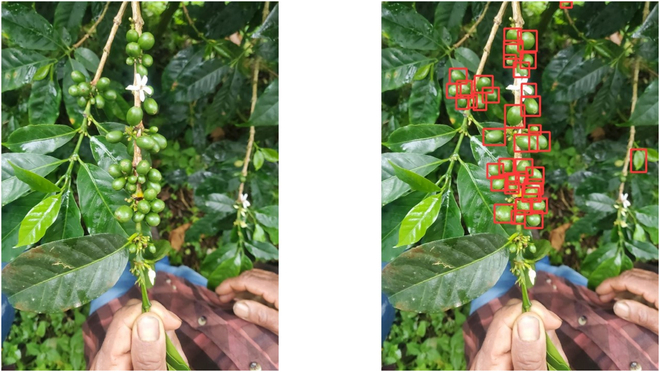
Green cherry detection using YOLO v8. This figure presents an example of detecting green cherries on a branch with YOLO v8. The detections are highlighted by red boxes, illustrating the precise identification of coffee cherries.

Figure 5 shows the comparison between manual counting and picture-based estimations for each branch within the dataset collected from Peru and Colombia. The highest correlation in measurement was observed in the lower position (branch 3) with an (*R*^2^ = 0.68), whereas the lowest correlation was noted in the upper position (branch 1) with an (*R*^2^ = 0.59). Figure 6 provides an example showcasing the capability of YOLO v8 in detecting cherries on branches, particularly emphasizing its high precision in detecting green cherries.

### Model performance at tree level

**Fig. 7.**
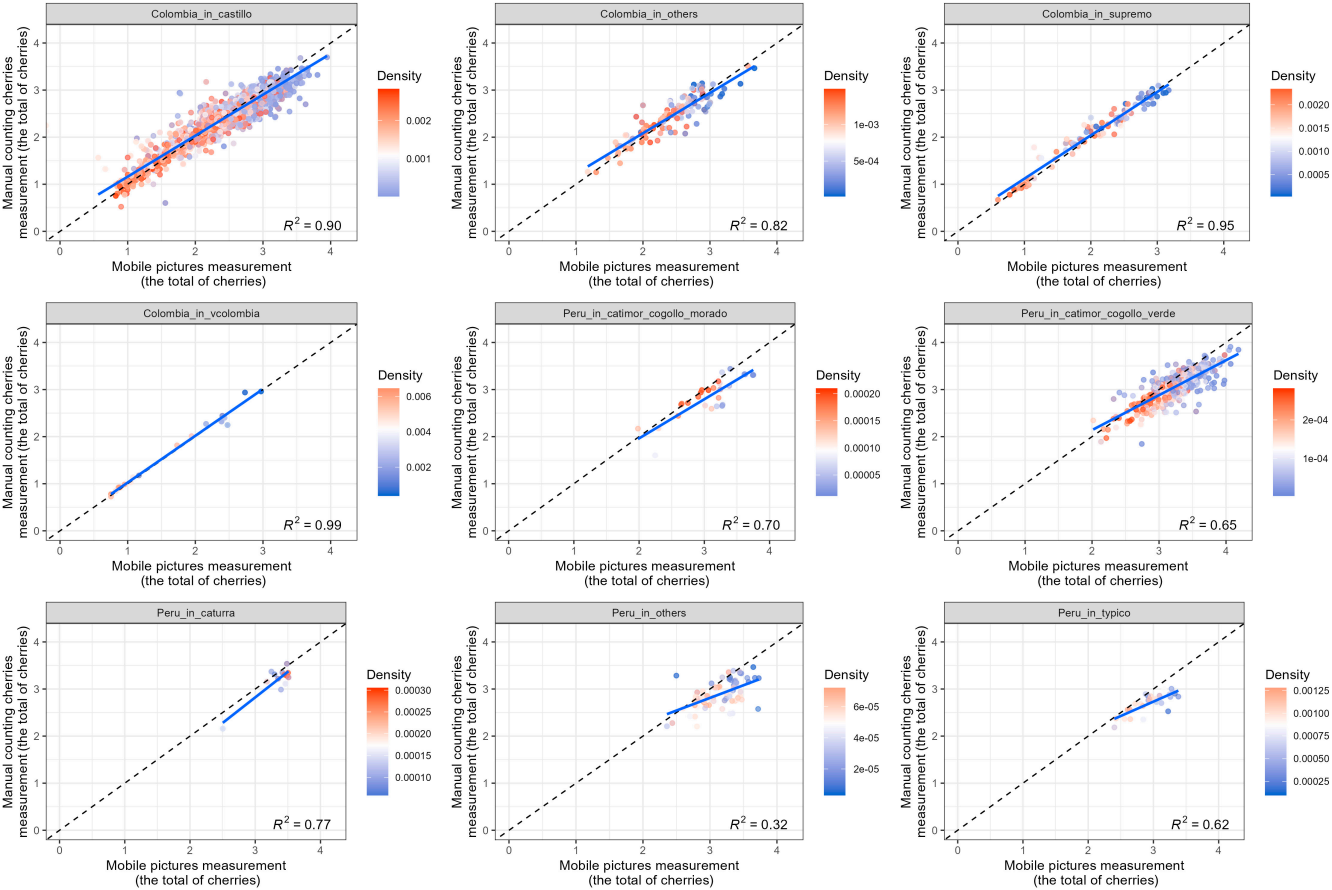
Predicted vs. measured number of coffee cherries per tree. This figure is a scatterplot comparing the predicted and actual number of coffee cherries per tree, categorized by country and variety. The axes are log10(X+1)-transformed.

We explore the effectiveness of our model across different coffee varieties and countries at the individual tree level. The model was initially trained using data from tree varieties such as *Catimor Cogollo Morado*, and *Catimogor Cogollo Verde* in Peru. Subsequently, we conducted tests in Colombia, evaluating its performance on varieties including *Castillo*, *Variedad Colombia*, *Supremo*, and others (Fig. 7).

The *R*^2^ model performance in Colombia was 0.71, demonstrating variability across different coffee varieties, with scores ranging from 0.82 to 0.99. The *Variedad Colombia* variety exhibited the highest performance. Notably, this surpasses the performance in Peru, where the model achieved an *R*^2^ of 0.59. In Peru, model performance varied among varieties, from 0.32 to 0.77, with the *Caturra* variety achieving the highest performance (Fig. 7). When we combined all test data from Colombia and Peru, the overall model's performance was as follows: the *R*^2^ of 0.72, the mean absolute error of 252.63 cherries per tree, the MAPE of 41.74 %, and the root mean square error of 621.46 cherries per tree.

## Discussion

We demonstrated the high performance (*R*^2^ = 0.72) of the first geographic-scale, low-cost method for monitoring coffee cherries using pictures taken with smartphones with an object detection model. The results showed that the vast majority of cherries were detected, similar to the findings in [23] which used YOLO v3 for apple detection, and [41] on bananas using the DeepLab with CNN. Our model´s performance was robust (*R*^2^ = 0.72, MAPE = 41.74%) and comparable to the methods proposed by [17] (*R*^2^ = 0.66), the branch-level machine vision system [42] (*R*^2^ = 0.93), deep learning combined with UAV imagery [16] (MAPE = 31.75%), and manual counting through quadrant-based sampling [9] (*R*^2^= 0.92).

The key advancement we achieved was the geographic extension of coffee cherry counting and monitoring, which has mostly been investigated at the field scale, without sacrificing predictive power. This study provides a marked contribution to a geographic-scale in situ phenotypic monitoring for coffee crops in low-income countries, which is scalable in space and time and thus helpful for monitoring the large-scale impacts of climate change on coffee crops. It can provide coffee cherry count data in regions lacking such information [42]. This information is valuable at a research level, as it can serve as input for other endeavors, such as creating strategies for climate change adaptation.

Our approach is notably promising because it diverges from prior research [16,17,42] by testing the model's efficacy in Colombia, distinct from its training environment in Peru. Notably, the model's performance improved, escalating from 0.59 in Peru to 0.71 in Colombia. The dissimilarities in coffee tree characteristics, geographical elevation, and climate conditions between the 2 countries were substantial, encompassing different species and varieties along with varying average cherry counts. Furthermore, in contrast to earlier studies [7,16,17], our data collection process utilized the efforts of local farmers, avoiding specialized equipment like multispectral cameras and drones. The photographs were taken by actual users with their conventional mobile phones. The object detection model, initially trained on *Catimor cogollo verde* and *Catimor cogollo morad*o varieties in Peru, was subsequently applied to count cherries in the *Tipica*, *Castillo*, *Supremo,* and the *variedad Colombia* varieties in Colombia. This approach highlights not only the model's adaptability but also its ability to generalize across different tree types in diverse agricultural settings. To further enhance model performance in a new environment, fine-tuning the developed deep learning model with images collected locally could be considered.

The model exhibits both overestimation and underestimation effects. The detection tends to overestimate the number of cherries in branches with few cherries, possibly due to the larger space captured in the picture, which allows for the inclusion of cherries from adjacent coffee plants. The coffee plant's challenging isolation, attributed to dimorphic branching [43] and the close proximity between plants, contributes to this effect. Conversely, the model demonstrates a tendency to underestimate, possibly influenced by the constrained space within mobile images or situations where only a portion of the branch is visible in the photo. This underestimation becomes noticeable when the count exceeds one thousand cherries, suggesting that the maximum number of cherries that can fit within a single mobile picture is around 1000.

The number of tree branches alone may not be sufficient for an accurate estimation of the total real number of cherries. Eight or 9 productive branches on Arabica plants could result in a real yield estimate of up to *R*^2^ = 0.92 [10]. However, at the plot level, this would require a large number of branches, making it impractical due to the unavailability of a large number of pictures and the time-consuming nature of the process. Moreover, there may be constraints related to connectivity and infrastructure that prevent the implementation of this approach on certain coffee farms.

The productive branches play a crucial role in our model. This result is consistent with findings of nondestructive methods [8,37]. Counting the number of productive branches is conducted manually, as the object detection model, YOLO v8, is not suitable due to the concealment of productive branches beneath large leaves. Adult coffee trees exhibit a leaf area ranging 22 and 45 m^2^ [37]. Although manual counting of branches may take much less time than counting cherries, we consider this task needs to be also supplemented with an alternative approach in the subsequent steps.

After evaluating the models, we realized that the design of a good photo-sampling protocol was crucial for accurately predicting the total number of cherries and thus advancing our monitoring approach. In Peru, many images were captured of whole trees rather than individual branches. These findings underscore the importance of following established protocols for image acquisition. Our protocol involves taking 3 pictures per tree, capturing the upper, middle, and lower branches, to capture the potential total number of cherries. Ensuring that these pictures accurately reflect the cherry quantity on the tree is paramount. Although our sampling protocol showed some promising results, there is still a large room for improvement in data acquisition. For instance, determining the optimal angles to capture most of the cherries will be crucial. The lower and middle branches may benefit from an overhead perspective, while the upper branch might require an underneath angle. Factors such as the direction of the sunlight, image blur, distortion (resulting from swift camera movement), and distance from the branches can also affect accuracy.

Identifying the main sources of error and refining the protocol can increase precision, but it should be noted that adding more photos or complexity may make local farmers more reluctant to use the method for their real-world application. The best approach between simplicity and accuracy needs to be identified with local farmers. In addition, future studies could focus on how to reliably scale the number of total cherries estimates from individual tree level to plot level yields, including the exploration of new species such as Robusta (*C. canephora*).

## Data Availability

Some representative pictures and the Python script used for the study are available at the GitHub repository: https://github.com/j-river1/Croppie.
